# The effect of prehypertension and hypertension on the incidence of cardiovascular disease: A population‐based cohort study in Kharameh, a city in the South of Iran

**DOI:** 10.1002/hsr2.1264

**Published:** 2023-05-25

**Authors:** Leila Moftakhar, Abbas Rezaianzadeh, Mozhgan Seif, Masoumeh Ghoddusi Johari, Seyed Vahid Hosseini, Seyed Sina Dehghani

**Affiliations:** ^1^ Student Research Committee Shiraz University of Medical Sciences Shiraz Iran; ^2^ Colorectal Research Center Shiraz University of Medical Science Shiraz Iran; ^3^ Department of Epidemiology, School of Health, Faculty of Biostatistics Shiraz University of Medical Sciences Shiraz Iran; ^4^ Breast Diseases Research Center Shiraz University of Medical Sciences Shiraz Iran; ^5^ School of Medicine Shiraz University of Medical Sciences Shiraz Iran

**Keywords:** cardiovascular diseases, cohort, hypertension, incidence, Iran, prehypertension

## Abstract

**Background and Aim:**

Prehypertension and hypertension are important risk factors for cardiovascular diseases. This study was carried out to evaluate the effect of prehypertension and hypertension on the development of cardiovascular diseases.

**Methods:**

This prospective cohort study was performed on 9442 people aged 40−70 in Kharameh, southern Iran. Individuals were divided into three groups: normal blood pressure (*N* = 5009), prehypertension (*N* = 2166), and hypertension (*N* = 2267). In this study, demographic data, disease histories, behavioral habits, and biological parameters were studied. At first, the incidence density was calculated. Then Firth's Cox regression models were used to investigate the association between prehypertension and hypertension with the incidence of cardiovascular diseases.

**Results:**

The incidence density in the three groups of individuals with normal blood pressure, prehypertension, and hypertension was 1.33, 2.02, and 3.29 cases per 100,000 person‐days, respectively. The results of multiple Firth's Cox regression by controlling all factors showed that the risk of occurrence of cardiovascular disease in people with prehypertension was 1.33 times (hazard ratio [HR] = 1.32, 95% confidence interval [CI]: 1.01−1.73, *p* = 0.03) and those with hypertension were 1.85 times higher (HR = 1.77, 95% CI: 1.38−2.29, *p* < 0.0001) than the individuals with normal blood.

**Conclusion:**

Prehypertension and hypertension have played an independent role in the risk for developing cardiovascular diseases. Therefore, early detection of individuals with these factors and control of other risk factors in them can contribute to reducing the occurrence of cardiovascular diseases.

## 1 INTRODUCTION

Cardiovascular diseases (CVDs) are the leading cause of morbidity and mortality worldwide.^1^ The estimated number of patients and deaths caused by these diseases was 485.6 and 17.7 million annually, respectively.^2^ Reports of the Global Burden of Disease (GBD) in 2019 showed that the number of patients and deaths due to CVDs doubled between 1990 and 2019.^3^ Iran was introduced as one of the countries with the highest number of CVDs in the world due to the GBD report in 2015.^4^

The most important preventive approaches for CVDs are changes in modifiable risk factors, including diabetes, hypertension, smoking, dyslipidemia, and low level of physical activity.^1^ Changing these risk factors could reduce CVDs by approximately 90% around the world.^4^ However, worldwide, Hypertension is an important risk factor for CVD, and it is the most prevalent risk factor. Tran et al.,^5^ The World Health Organization reported the number of people with hypertension to be 1.13 and 1.27 million deaths from CVDs attributed to hypertension billion (https://www.who.int/health-topics/hypertension#tab=tab_1). In Iran, the prevalence of hypertension among people aged 40−70 years is 26.9%, and the risk attributed to hypertension for CVDs is estimated at 11.37%.^4^

So far, several epidemiological studies have investigated the association between prehypertension and hypertension with CVDs in other parts of the world, that their results are contradictory.^2^, ^6^ However, there is currently no updated information on the subject. In addition, cohort studies that provide robust evidence are few in this regard. Furthermore, in a literature review, we found no cohort studies that examined the association between prehypertension and hypertension with the incidence of CVDs in Iran. Meanwhile, having new and up‐to‐date information from different regions of the world and identifying the exact role of prehypertension and hypertension in CVDs help health policymakers plan to develop interventional strategies for these people. Therefore, this study was conducted with a large sample size, a large number of risk factors compared to other studies, and by using the Firth's Cox regression model to reduce the error of estimates due to rare events, to accurately identify the role of hypertension and prehypertension in the occurrence of CVDs.

## 2 METHODS

### 2.1 Study population

This prospective study was based on the data from the Kharameh cohort study, which is a part of Prospective Epidemiological Studies in Iran (PERSIAN). The Persian cohort study was launched in 2014 to determine the prevalence and risk factors associated with noncommunicable diseases (NCDs) in 18 areas of Iran. Additional information are provided in other articles.^7^ Kharameh is a city located in the southern region of Fars province. The people of this city have Persian ethnicity and race, and they also have customs and cultural traditions specific to their region. In addition, the lifestyle in the urban and rural areas of this city is somewhat similar. These characteristics have distinguished them from others areas. The Kharameh cohort study was launched with a population of 10,663 individuals aged 40−70 years in 2015 to determine risk factors for NCDs at the time of the baseline survey and during the follow‐up period. 93.7% of people between the ages 40−70 who living in Kheramah participated in this study and gave informed consent.

**Figure 1:**
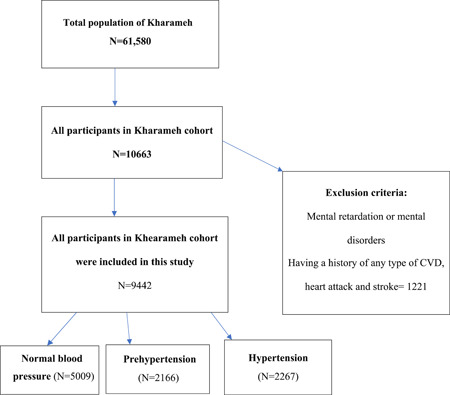
Flow chart of the study population.

The inclusion and exclusion criteria for the Kharamah cohort study are stated in another study. Baeradeh et al.,^8^ exclusion criteria for this study included having a history of CVDs, heart attack, and stroke. Accordingly, 1221 individuals were excluded from the study, and finally, 9442 of them remained in the study; they were divided into three categories: the individuals with normal blood pressure (*N* = 5009), with prehypertension (*N* = 2166), and with hypertension (*N* = 2267) (Figure 1).

### 2.2 Data collection

The baseline data of the Kharameh cohort study were collected from March 2015 to March 2017, and, the information about the incidence of CVD in individuals has been collected during four stages of follow‐up, in 2018, 2019, 2020, and 2021. Questionnaires from the Persian cohort study, which had already been validated, were used for data collection. The information related to these questionnaires as well as the data collection method is included in the Persian cohort study.^7^

This study examined demographic information, behavioral habits, history of chronic diseases, anthropometric characteristics, and some biological parameters. The demographic information included age, sex, job status, marital status, place of residence, level of education, and socioeconomic status (SES). The Persian cohort questionnaires related to the individuals' assets were completed to assess their SES. Physical activity levels were also assessed using standard questionnaires that measure daily activity. The calculation of the SES of people and the level of physical activity is also described in other studies of the Kharamah cohort.^9^, ^10^

Behavioral habits also included levels of physical activity, smoking, alcohol consumption, and anthropometric characteristics, including body mass index (BMI), waist circumference, and hip circumference. A history of diabetes, fatty liver, and chronic kidney disease was also evaluated. In addition, biological parameters, including low‐density lipoprotein (LDL) and triglyceride (TG), were assessed.

BMI was calculated by dividing weight (kg) by height in square meters (m^2^), and individuals were divided into four classes. Underweight: BMI less than 18.5; normal: BMI between 18.5 and 24.9; overweight: BMI between 25 and 29.9; and obese: BMI above 30.^11^

### 2.3 Measurement and classification of blood pressure

Blood pressure was measured on the right upper arm of the subject in a sitting position after 5 min of rest. Blood pressure was taken from each individual twice at 10 min, and their mean was recorded as blood pressure. All measurements were performed using a standard calibration sphygmomanometer. Individuals were divided into three groups: individuals with normal blood pressure: blood pressure of SBP/DBP less than 120/80 mmHg; prehypertension group: SBP between 120  and 139 mmHg or DBP between 80 and 89 mmHg; and hypertension group: SBP/DBP above 140/90 mm/Hg.^12^

### 2.4 CVDs

In this study, the subjects were followed‐up from the time of entry into the study to the time of the event or the end of the study. The incidence of CVDs was initially recorded using an interview based on their self‐declaration. Then, their medical records were checked by physicians, and if were confirmed, they were registered as new cases of CVDs. If necessary, more diagnostic procedures were performed. CVDs included coronary heart disease, cerebrovascular disease, rheumatoid arthritis, myocardial infarction, stroke, and heart valve disease.

### 2.5 Statistical analysis

In the present study, the dependent variable was the time to event of CVDs. The time variable was calculated as the number of days between entry into the study and the occurrence of CVDs. The participants were also considered the right censors if the event did not happen to them until the end of the study. Quantitative variables were described with mean and standard deviation, and qualitative variables were presented with numbers and percentages. The Kolmogorov−Smirnov test assessed the normality of quantitative variables. The difference between the means of quantitative variables in three groups of individuals was examined using the ANOVA test and the difference between the levels of qualitative variables was examined using the *χ* ^2^ test. The incidence density was calculated on the basis of the person‐day, which is the actual number of days individuals are at risk for CVDs. For the survival analysis, the Kaplan−Meier curve for the incidence of CVDs in the three groups of subjects was plotted. The log‐rank test was calculated to compare the incidence of CVDs between these three groups. Also, we evaluated the effect of prehypertension and hypertension on the incidence of CVDs using three models in Firth's Cox regression. In the first model, the effect of prehypertension and hypotension was evaluated without adjusting for other variables. The second model was adjusted for age and sex variables, and the third model was adjusted for all variables with a *p*‐value less than 0.2 in simple regression. The power of the association was reported with the hazard ratio (HR) with a 95% confidence interval (CI). All analyses were performed using R version 4.1.2, the “Coxphf” package.

## 3 RESULTS

### 3.1 Distribution of baseline information

The present study was performed on 9442 individuals aged 40−70 years. They were divided into three groups: normal blood pressure (*N* = 5009), prehypertension (*N* = 2166), and hypertension (*N* = 2267). Table 1 shows the comparison of the basic information in these three groups. The mean (SD) age of the subjects was 51.47 (8/05) years, which was significantly higher in the hypertension group than in the prehypertension and normal groups (54.8 [8.05] vs. 51.8 [7.88] and 49.7 [7.61] years, *p* < 0.0001). The mean (SD) TG in the hypertension group was significantly higher than in the other two groups (137.099 [79.58] vs. 132.02 [73.4] and 125.81 [73.49], *p* < 0.0001). Also, the prevalence of fatty liver and diabetes was also significantly higher in the hypertension group than in the other two groups (*p* < 0.0001). Further information is reported in Table 1.

**Table 1:** Distribution of baseline characteristics in three groups of individuals with prehypertension, hypertension, and normal blood pressure in a population aged 40−70 years of Kharameh cohort study.

Variable		Total (*N* = 9442)	Normal (*N* = 5009)	Prehypertension (*N* = 2166)	Hypertension (*N* = 2267)	*p*
		Mean ± SD	Mean ± SD	Mean ± SD	Mean ± SD	
Age (years)		51.47 ± 8.04	49.77 ± 7.6	51.84 ± 7.88	54.86 ± 8.05	0.0001^a^
TG		129.95 ± 80.49	125.81 ± 80.8	132.02 ± 80.09	137.09 ± 79.6	0.0001^a^
LDL		105.62 ± 31.46	104.75 ± 27.59	106.48 ± 26.98	106.69 ± 50.63	0.025^a^
Waist circumference		95.15 ± 12.03	92.74 ± 12.01	96.17 ± 11.34	99.52 ± 11.33	0.0001^a^
Hip circumference		100.83 ± 8.43	99.81 ± 8.31	101.41 ± 7.82	102.53 ± 8.91	0.0001^a^

	**Class**	***N* (%)**	***N* (%)**	***N* (%)**	***N* (%)**	***p***
Sex	Male	4192 (44.4)	2422 (48.35)	1087 (50.18)	683 (30.13)	0.0001^b^
	Female	5250 (55.6)	2587 (51.65)	1079 (49.82)	1584 (69.87)	
Employment	Unemployed	4400 (46.6)	2060 (41.13)	921 (42.52)	1419 (62.50)	0.0001^b^
	Employed	5042 (53.4)	2949 (87.87)	1245 (57.48)	848 (37.46)	
Married status	Single	166 (1.76)	100 (2)	39 (1.80)	27 (1.19)	0.0001^b^
	Married	8472 (89.73)	4605 (91.93)	1953 (90.17)	1914 (84.43)	
	Divorced	89 (0.94)	52 (1.04)	16 (0.74)	21 (0.93)	
	Widow	715 (7.57)	252 (5.03)	158 (7.29)	305 (13.45)	
Socioeconomic status	Low	2372 (25.12)	1234 (24.64)	565 (26.08)	573 (25.28)	0.796^b^
	Moderate	2636 (27.92)	1415 (28.25)	581 (26.82)	640 (28.23)	
	High	2229 (23.61)	1183 (23.62)	507 (23.41)	539 (23.87)	
	Very high	2205 (23.35)	1177 (23.50)	513 (23.68)	515 (22.72)	
Physical activity	Low	8517 (90.2)	4409 (88.02)	1951 (90.07)	2157 (95.15)	0.0001^b^
	Moderate	925 (9.8)	600 (11.98)	215 (9.92)	110 (4.85)	
Smoking	No	7083 (75.02)	3561 (71.09)	1599 (73.80)	1923 (84.83)	0.0001^b^
	Yes	2359 (24.98)	1448 (28.91)	567 (26.18)	344 (15.17)	
Alcohol consumption	No	8924 (94.51)	4685 (93.53)	2033 (93.86)	2206 (97.31)	0.0004^b^
	yes	518 (5.49)	324 (6.47)	133 (6.14)	61 (2.69)	
Living place	Urban	3379 (35.79)	1889 (37.71)	653 (30.15)	837 (36.92)	0.0001^b^
	Rural	6063 (64.21)	3120 (62.29)	1513 (69.8)	1513 (69.8)	
BMI	Under weight	380 (4.02)	283 (5.65)	71 (3.28)	26 (1.15)	0.0001^b^
	Normal	3493 (36.99)	2133 (42.58)	731 (33.75)	629 (27.75)	
	Over weight	3914 (41.45)	1819 (37.75)	994 (45.89)	1029 (45.39)	
	Obese	1655 (17.53)	702 (14.01)	370 (17.08)	583 (25.72)	
Chronic kidney disease	No	9411 (99.67)	5002 (99.86)	2159 (99.68)	2250 (99.25)	0.0001^b^
	Yes	31 (0.33)	7 (0.14)	7 (0.32)	17 (0.75)	
Fatty livre	No	8383 (88.87)	4544 (90.72)	1939 (89.52)	1900 (83.82)	0.0001^b^
	Yes	1059 (11.22)	465 (9.28)	227 (10.48)	367 (16.19)	
Diabetes mellitus	No	8182 (86.66)	4569 (91.22)	1933 (89.52)	1680 (74.11)	0.0001^b^
	Yes	1260 (13.34)	440 (8.78)	233 (10.76)	587 (25.89)	

Abbreviations: LDL, low‐density lipoprotein; TG, triglyceride.

^a^ ANOVA test.

^b^ *χ* ^2^ test.

### 3.2 Duration of follow‐up and occurrence of CVDs

In this study, the subjects were followed for 10,514,688 person‐days in the normal blood pressure group, for 4,586,820 person‐days in the prehypertension group, and 4,643,446 person‐days in the hypertension group. The incidence density rate was estimated at 1.33 per 100,000 person‐days in the normal blood group, 2.02 per 100,000 person‐days in the prehypertension group, and 3.2 per 100,000 person‐days in the hypertension group (Table 2).

**Table 2:** Incidence of cardiovascular disease in three groups of individuals with prehypertension, hypertension, and normal blood pressure in a population aged 40−70 years of Kharameh cohort study.

	Cases of CVD	Follow‐up (person‐day)	Incidence (per 100,000 person‐day)
Normal blood pressure	140	10,514,688	1.33
Pre hypertension	93	456,820	2.02
Hypertension	153	4,643,446	3.2

Abbreviation: CVD, cardiovascular diseases.

**Figure 2:**
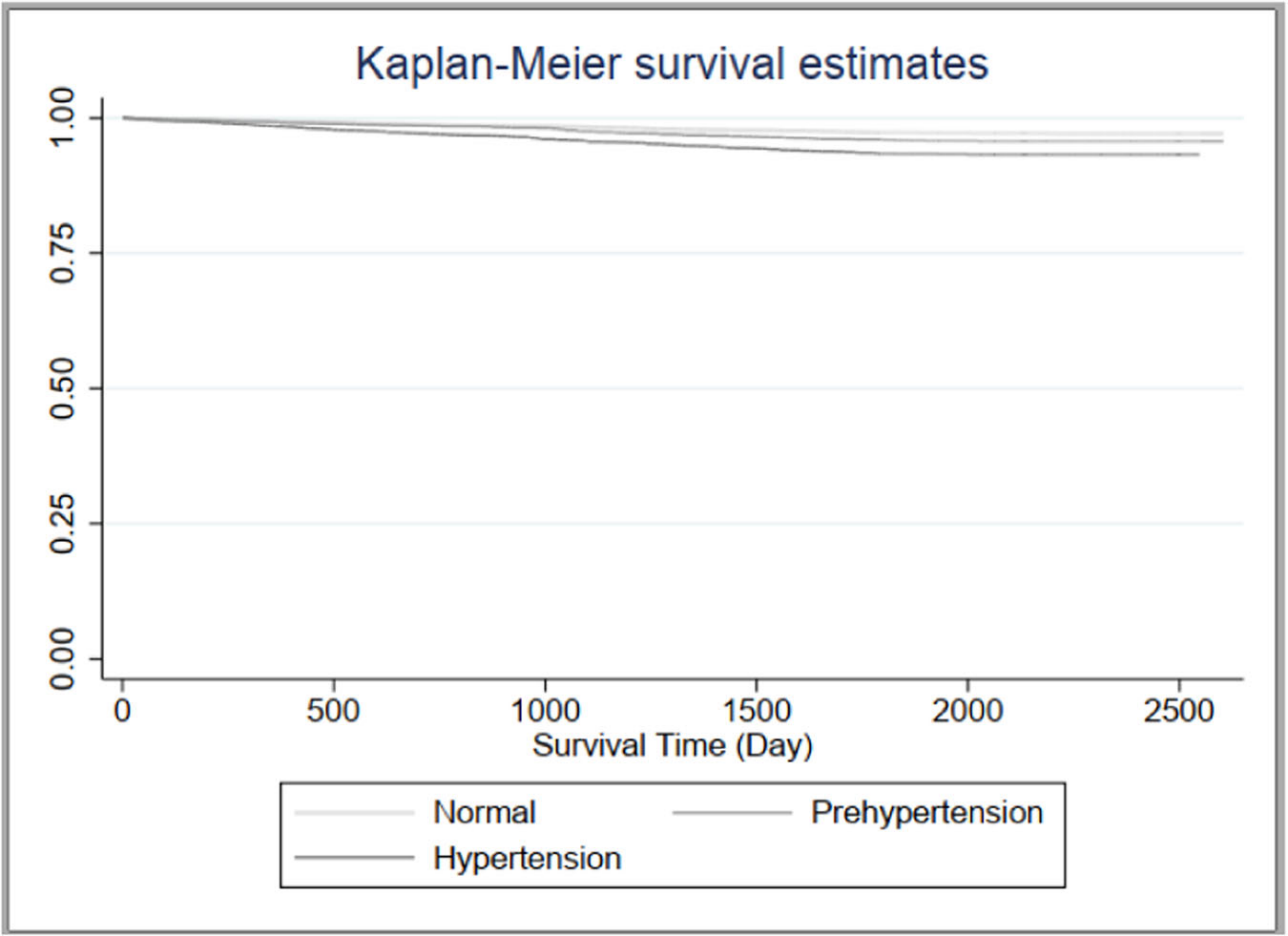
Kaplan−Meier survival curve in three groups of individuals with prehypertension, hypertension, and normal blood pressure.

Figure 2 shows the CVDs survival curve for the three groups. The log‐rank test showed that the risk of developing CVDs in the hypertension group was significantly higher than in the prehypertension and normal blood pressure groups (*χ* ^2^ = 63.03 *p* < 0.0001).

### 3.3 Association between prehypertension and hypertension with the occurrence of CVDs

In three models, the Firth's Cox regression model was performed to investigate the association between prehypertension and hypertension with the risk of developing CVDs. In the first model, the roles of prehypertension and hypertension were investigated without adjusting for other variables; the results showed that the risk of developing CVDs in the prehypertension group was 1.54 times (HR: 1.54 95% CI: 1.18−2.006, *p* = 0.001); in the hypertension group, it was 2.46 times (HR: 2.46 95% CI: 1.95−3.09, *p* < 0.0001) higher than the normal blood pressure group. In model 2, after adjusting for age and sex, with a slight reduction, the risk of developing CVDs in the prehypertension group was 1.31 times (HR = 1.31, 95% CI: 1.007−1.7, *p* = 0.04); in the hypertension group, it was 1.98 times higher (HR = 1.98, 95% CI: 1.56−2.53, *p* < 0.0001) compared with the normal blood pressure group. The third model was adjusted for several variables, including age, sex, job status, marital status, level of physical activity, hip circumference, smoking, alcohol consumption, diabetes, and chronic kidney disease. This model had a *p*‐value less than 0.2 in the simple Firth‐Cox regression model. Finally, the results showed that the risk of developing CVDs in the prehypertension group was 1.33 times (HR = 1.32, 95% CI: 1.01−1.73, *p* = 0.03) and in the hypertension group 1.85 times (HR = 1.77, 95% CI: 1.38−2.29, *p* < 0.0001) higher than that of the normal blood pressure group (Table 3).

**Table 3:** The association between prehypertension and hypertension with incidence of cardiovascular disease based on the result Firth's Cox regression in a population aged 40−70 years of Kharameh cohort study.

	Model 1		Model2		Model3	
	**HR (95% CI)**	***p*** a	**HR**_**adj** _ **(95% CI)**	***p*** a	**HR**_**adj** _ **(95% CI)**	***p*** a
Normal	1		1		1	
Prehypertension	1.54 (1.18−2.006)	0.001	1.31 (1.007−1.7)	0.04	1.32 (1.01−1.73)	0.03
Hypertension	2.46 (1.95−3.09)	0.0001	1.98 (1.56−2.53)	0.0001	1.77 (1.38−2.29)	0.0001

*Note*: Model 1: Simple Cox regression; Model2: Cox regression adjusted for age and sex, prehypertension, hypertension; Model3: Multiple Cox regression adjusted for some variables, including age, sex, job status, marital status, level of physical activity, TG, hip circumference, smoking, alcohol consumption, diabetes, and chronic kidney disease, prehypertension, hypertension.

^a^ Firth's Cox regression.

## 4 DISCUSSION

The purpose of this study was to study the role of prehypertension and hypertension in the risk of developing CVDs among 9442 individuals aged 40−70 years old in Kharameh (a city in the south of Iran). The incidence density of CVDs was 2.02 and 3.29 cases per 100,000 person‐days in those with prehypertension and hypertension, respectively.

This study showed that the risk of developing CVDs in prehypertensive and in hypertensive individuals was higher than in those with normal blood pressure. Numerous epidemiological studies have examined the association between prehypertension and hypertension with the risk of developing CVDs. However, some of these studies have not shown an association between prehypertension and hypertension with the incidence of CVDs,^13^, ^14^, ^15^, ^16^ while some have shown a direct association.^6^, ^17^, ^18^, ^19^, ^20^, ^21^ A cohort study conducted in China, after 4 years of follow‐up, revealed that the incidence of CVDs in individuals with prehypertension was 1.19 higher than that in subjects with normal blood pressure; After adjusting for other variables, prehypertension has been shown to increase the risk of developing CVDs by 1.32 times.^22^ Haung et al. also found that individuals with prehypertension were 1.5 times more likely to develop CVDs.^18^ Guoju Li et al. reported a dose–response association between high blood pressure levels and the incidence of CVDs.^23^ Generally, increasing blood pressure levels to prehypertension and hypertension increases the stress cycle on the main arteries, resulting in increased wear and tear of these arteries earlier, eventually leading to CVDs. However, the exact mechanism of the role of blood pressure in the development of CVDs remains unclear. Some studies suggest that hypertension has disrupted the regulation of the renin‐angiotensin and aldosterone systems through increased adiposity. For this reason, adipokines and leptins increase and lead to the hardening of the arteries, eventually, the incidence of CVD increases. On the other hand, prehypertension causes deformation of the left ventricle and a disorder of diastolic blood pressure, which ultimately increases the incidence of CVDs.^24^

It is also important to note that although the role of prehypertension and hypertension is recognized as an independent risk factor for the development of CVDs, if other risk factors accompany these factors, they increase the risk of developing CVDs more than when there is one factor alone.^25^ However, many studies have shown that high BMI, LDL, TG, age, diabetes, and other risk factors for CVDs are more common in individuals with prehypertension and hypertension than in those with normal blood pressure.^6^, ^22^ In our study, the mean age of hypertensive was higher than prehypertensive patients and normal blood pressure subjects. Mean TG, LDL, and waist circumference were also higher among the hypertensive, prehypertensive subjects, and those with normal blood pressure, respectively. The prevalence of diabetes was also higher in hypertensive individuals. In addition, the prevalence of obesity and overweight was 68% in individuals with hypertension, 62% in individuals with prehypertension, and 52% in individuals with normal blood pressure. A cohort study conducted in China also showed that mean of TG, LDL, and BMI were higher in individuals with hypertension than those with normal blood pressure.^22^ In Ajab Noor et al.'s study, subjects with hypertension and prehypertension compared to normal blood pressure had higher risk factors for CVDs, such as BMI, LDL, TG, and cholesterol. However, the association between blood pressure and CVDs can be reduced by modifying several risk factors.^25^ Therefore, effective interventions in this field will significantly help to reduce the incidence of CVDs.

### 4.1 Strengths and limitations

This is a prospective study with a large sample size that provides stronger evidence for investigating causality than retrospective and cross‐sectional studies. We also adjusted a wide range of variables to investigate the independent role of prehypertension and hypertension in the incidence of CVDs. One of the limitations of the present study is the lack of access to some risk factors, such as people's nutritional status, environmental factors, and air pollution, which can play a role in the occurrence of CVDs. In addition, we did not have the data of various types of CVDs separately, which can also affect our results, because the role of prehypertension and hypertension on various types of CVDs can be different. In addition, the follow‐up period of our study was short,

## 5 CONCLUSION

The results of this study showed that prehypertension and hypertension are independent and relatively strong risk factors for the development of CVDs. Therefore, due to the high prevalence of prehypertension and hypertension and other risk factors of CVDs in these individuals, effective interventions to control these risk factors would substantially reduce the incidence of CVDs. Lifestyle modification education for people with prehypertension and hypertension s one of the most effective interventions. Also, identifying high‐risk individuals with hypertension and prehypertension and performing interventions on these individuals, their timely treatment, blood pressure control, and modification of modifiable risk factors play an important role in reducing the incidence of CVDs in these people.

## AUTHOR CONTRIBUTIONS

**Leila Moftakhar**: Conceptualization; investigation; writing—original draft. **Abbas Rezaianzadeh**: Conceptualization; supervision. **Mozhgan Seif**: Data curation; formal analysis. **Masoumeh Ghoddusi Johari**: Software; writing—original draft. **Seyed Vahid Hosseini**: Writing—original draft. **Seyed Sina Dehghani**: Conceptualization; investigation. All authors have read and approved the final version of the manuscript.

## CONFLICT OF INTEREST STATEMENT

The authors declare no conflict of interest.

## ETHICS STATEMENT

PERSIAN Cohort Study is being performed in 18 geographical regions of Iran. PERSIAN Cohort Study was approved by the Ethics Committee of the Ministry of Health and Medical Education. This study was in agreement with the Helsinki Declaration and Iranian national guidelines for ethics in research. This research is extracted from a PhD dissertation under the supervision of Dr. Abbas Rezaianzadeh. It has also been approved by the ethics committee of Shiraz University of Medical Sciences (IR.SUMS.SCHEANUT.REC.1400.046). Besides, written informed consent was obtained from all the participants.

## TRANSPARENCY STATEMENT

The lead author Abbas Rezaianzadeh affirms that this manuscript is an honest, accurate, and transparent account of the study being reported; that no important aspects of the study have been omitted; and that any discrepancies from the study as planned (and, if relevant, registered) have been explained.

## Data Availability

The data sets used and/or analyzed during the current study are available from the corresponding author upon reasonable request. Abbas Rezaianzadeh had full access to all of the data in this study and takes complete responsibility for the integrity of the data and the accuracy of the data analysis.

## References

[1] Khosravi A , Gharipour M , Nezafati P , et al. Pre‐hypertension, pre‐diabetes or both: which is best at predicting cardiovascular events in the long term? J Hum Hypertens. 2017;31:382‐387. 10.1038/jhh.2016.42 27334522

[2] Han M , Li Q , Liu L , et al. Prehypertension and risk of cardiovascular diseases: a meta‐analysis of 47 cohort studies. J Hypertens. 2019;37:2325‐2332. 10.1097/HJH.0000000000002191 31335511

[3] Roth GA , Mensah GA , Johnson CO , et al. Global burden of cardiovascular diseases and risk factors, 1990–2019. JACC. 2020;76:2982‐3021. 10.1016/j.jacc.2020.11.010 33309175 PMC7755038

[4] Sarrafzadegan N , Mohammadifard N . Cardiovascular disease in Iran in the last 40 years: prevalence, mortality, morbidity, challenges and strategies for cardiovascular prevention. 2019. 31126179

[5] Tran D‐MT , Lekhak N , Gutierrez K , Moonie S . Risk factors associated with cardiovascular disease among adult Nevadans. PLoS ONE. 2021;16:e0247105. 10.1371/journal.pone.0247105 33596242 PMC7888645

[6] Ajabnoor GMA , Bahijri S , Alamoudi AA , et al. The association between hypertension and other cardiovascular risk factors among non‐diabetic Saudis adults—a cross sectional study. PLoS ONE. 2021;16:e0246568. 10.1371/journal.pone.0246568 33621259 PMC7901777

[7] Poustchi H , Eghtesad S , Kamangar F , et al. Prospective epidemiological research studies in Iran (the PERSIAN Cohort Study): rationale, objectives, and design. Am J Epidemiol. 2018;187:647‐655. 10.1093/aje/kwx314 29145581 PMC6279089

[8] Baeradeh N , Ghoddusi Johari M , Moftakhar L , Rezaeianzadeh R , Hosseini SV , Rezaianzadeh A . The prevalence and predictors of cardiovascular diseases in Kherameh cohort study: a population‐based study on 10,663 people in Southern Iran. BMC Cardiovasc Disord. 2022;22:244. 10.1186/s12872-022-02683-w 35643460 PMC9148515

[9] Moftakhar L , Jafari F , Ghoddusi Johari M , Rezaeianzadeh R , Hosseini SV , Rezaianzadeh A . Prevalence and risk factors of kidney stone disease in population aged 40–70 years old in Kharameh cohort study: a cross‐sectional population‐based study in Southern Iran. BMC Urol. 2022;22:205. 10.1186/s12894-022-01161-x 36536352 PMC9764470

[10] Moftakhar L , Rezaeianzadeh R , Ghoddusi Johari M , Hosseini SV , Rezaianzadeh A . Epidemiology and predictors of multimorbidity in Kharameh cohort study: a population‐based cross‐sectional study in Southern Iran. Health Science Reports. 2023;6:e988. 10.1002/hsr2.988 36514331 PMC9731168

[11] Organization, W. H . Obesity: preventing and managing the global epidemic. Report of a World Health Organization Consultation. World Health Organization. WHO obesity technical report series, 2000;284:256. 11234459

[12] Arima H , Murakami Y , Lam TH , et al. Effects of prehypertension and hypertension subtype on cardiovascular disease in the Asia‐Pacific region. Hypertension. 2012;59:1118‐1123. 10.1161/HYPERTENSIONAHA.111.187252 22547441

[13] Hozawa A , Kuriyama S , Kakizaki M , Ohmori‐Matsuda K , Ohkubo T , Tsuji I . Attributable risk fraction of prehypertension on cardiovascular disease mortality in the Japanese population: the Ohsaki Study. Am J Hypertens. 2009;22:267‐272. 10.1038/ajh.2008.335 19039309

[14] Ikeda A , Iso H , Yamagishi K , Inoue M , Tsugane S . Blood pressure and the risk of stroke, cardiovascular disease, and all‐cause mortality among Japanese: the JPHC study. Am J Hypertens. 2009;22:273‐280. 10.1038/ajh.2008.356 19229210

[15] Mainous III, AG , Everett CJ , Liszka H , King DE , Egan BM . Prehypertension and mortality in a nationally representative cohort. Am J Cardiol. 2004;94:1496‐1500. 10.1016/j.amjcard.2004.08.026 15589003

[16] Sairenchi T , Iso H , Irie F , et al. Age‐specific relationship between blood pressure and the risk of total and cardiovascular mortality in Japanese men and women. Hypertension Res. 2005;28:901‐909. 10.1291/hypres.28.901 16555579

[17] Guo X , Zhang X , Guo L , et al. Association between pre‐hypertension and cardiovascular outcomes: a systematic review and meta‐analysis of prospective studies. Curr Hypertens Rep. 2013;15:703‐716. 10.1097/HJH.0000000000002191 24234576

[18] Huang Y , Wang S , Cai X , et al. Prehypertension and incidence of cardiovascular disease: a meta‐analysis. BMC Med. 2013;11:177. 10.1186/1741-7015-11-177 23915102 PMC3750349

[19] Kalaitzidis RG , Bakris GL . Prehypertension: is it relevant for nephrologists? Kidney Int. 2010;77:194‐200. 10.1038/ki.2009.439 19924105

[20] Lewington S , Prospective Studies Collaboration . Age‐specific relevance of usual blood pressure to vascular mortality: a meta‐analysis of individual data for one million adults in 61 prospective studies. Lancet. 2002;360:1903‐1913. 10.1016/S0140-6736(02)11911-8 12493255

[21] Shen L , Ma H , Xiang M‐X , Wang J‐A . Meta‐analysis of cohort studies of baseline prehypertension and risk of coronary heart disease. Am J Cardiol. 2013;112:266‐271. 10.1016/j.amjcard.2013.03.023 23608614

[22] Wu S , Huang Z , Yang X , et al. Cardiovascular events in a prehypertensive Chinese population: four‐year follow‐up study. Int J Cardiol. 2013;167:2196‐2199. 10.1016/j.ijcard.2012.05.123 22805539

[23] Li G , Guo G , Wang W , et al. Association of prehypertension and cardiovascular risk factor clustering in Inner Mongolia: a cross‐sectional study. BMJ Open. 2017;7:e015340. 10.1136/bmjopen-2016-015340 PMC5734362 28667215

[24] Duan W , Wu J , Liu S , et al. Impact of prehypertension on the risk of major adverse cardiovascular events in a Chinese rural cohort. Am J Hypertens. 2020;33:465‐470. 10.1093/ajh/hpaa019 32030405

[25] Kjeldsen SE . Hypertension and cardiovascular risk: general aspects. Pharmacol Res. 2018;129:95‐99. 10.1016/j.phrs.2017.11.003 29127059

